# Aerobic Exercise Improves Pulmonary Fibrosis by Improving Insulin Resistance and Inflammation in Obese Mice

**DOI:** 10.3389/fphys.2021.785117

**Published:** 2022-01-18

**Authors:** Xishuai Wang, Xuejie Yi, Donghui Tang

**Affiliations:** ^1^Department of College of P.E. and Sports, Beijing Normal University, Beijing, China; ^2^Department of Animal Genetic Resources, Institute of Animal Science, Chinese Academy of Agricultural Sciences, Beijing, China; ^3^Department of Kinesiology, Shenyang Sport University, Shenyang, China

**Keywords:** obesity, pulmonary fibrosis, inflammation, oxidative stress, insulin resistance, exercise

## Abstract

**Background:**

Previous studies have demonstrated that obesity is associated with pulmonary fibrosis. We attempted to identify whether regular aerobic exercise (AE) can protect against high-fat diet (HFD)-associated pulmonary fibrosis.

**Methods:**

Forty-eight C57BL/6 mice were randomly assigned to four groups: chow group (Ch), chow plus exercise group (CE), obesity group (Ob), and obesity plus exercise group (OE). The mice were fed either an HFD or a chow diet for 16 weeks, and low-intensity aerobic exercise (AE) was performed in the last 8 weeks. We measured the degree of pulmonary fibrosis; pulmonary inflammation; oxidative stress parameters; insulin resistance-related indicators; the number of inflammatory cells in bronchoalveolar lavage fluid (BALF); the mRNA expression levels of IL-10, IL-1β, TGF-β, TNF-α, CXCL-1, IL-17, MMP-9, MPO, NE, and sirt-1; and the BALF levels of CXCL-1, IL-17, TGF-β, IL-10, IL-1β, and TNF-α in lung tissue.

**Results:**

AE in obese mice protected against obesity-associated pulmonary fibrosis, chronic inflammation, pro-oxidative/antioxidative imbalance, and insulin resistance. AE ameliorated the HFD-induced inflammatory response and neutrophil infiltration in the lung. AE downregulated BALF levels of CXCL-1, IL-1β, TNF-α IL-17, and TGF-β but upregulated BALF levels of IL-10. AE decreased IL-1β, TGF-β, TNF-α, CXCL-1, IL-17, MMP-9, MPO, and NE mRNA expression levels but upregulated IL-10 and sirt-1 mRNA expression levels in the lung.

**Conclusions:**

AE protects against HFD-induced pulmonary fibrosis by improving obesity-associated insulin resistance, chronic low-grade inflammation, and pro-oxidative/antioxidative imbalance. AE improved HFD-induced pulmonary fibrosis by suppressing IL-17, TGF-β, NE, and MMP-9 expression and activating IL-10 and sirt-1 expression.

## Introduction

The prevalence of obesity is increasing drastically and its prevalence markedly upregulates the incidence of many complications such as chronic obstructive pulmonary disease (COPD), asthma, and pulmonary fibrosis (Bianco et al., 2017; Liu et al., 2017; Murtha et al., 2017; Rathinasabapathy et al., 2018; Bluher, 2019). At present, pulmonary fibrosis is a deadly chronic interstitial disease and a substantial cause of morbidity worldwide.

Obesity results in insulin resistance, vitamin D deficiency, and increased expression of fibrogenic factors including TGF-β, and proinflammatory cytokines such as TNF-α and C-reactive protein; these changes cause pulmonary fibrosis (Wortsman et al., 2000; Ford et al., 2005; Botella-Carretero et al., 2007; Vimaleswaran et al., 2013; Han et al., 2021). The vitamin D receptor (VDR) exists in bronchial epithelial cells and is an important cause of pulmonary fibrosis partly because vitamin D deficiency can regulate TGF-β signalling by suppressing phosphorylated Smad-2/3 and activating the renin-angiotensin system (RAS) activity (Li et al., 2004; Adav et al., 2011; Zerr et al., 2015; Shi et al., 2017; Park et al., 2018; Tzilas et al., 2019). Insulin resistance mediates pulmonary fibrosis *via* the TGF-β pathway (Park et al., 2019). *In vitro* experiments have demonstrated that bronchial cells and cells from insulin-resistant subjects were associated with increased TGF-β activation and collagen deposition (Spencer et al., 2010; Mayer et al., 2012). Insulin resistance is closely associated with proinflammatory reactions involving inflammatory cells and inflammatory cytokines (Asghar and Sheikh, 2017). Obesity is known to lead to changes in inflammatory parameters and impaired oxidative capacity, which are important risk factors for and pathogenic mechanisms in pulmonary fibrosis (Nishiyama et al., 2005; Jackson et al., 2010, 2014). Inflammatory cytokines, including TNF-α and IL-1β, and immune cells, including neutrophils, are important causes of pulmonary fibrosis (Stockley, 2002; Thannickal et al., 2004). Neutrophil elastase (NE) and matrix metalloproteinase 9 (MMP-9) are released by neutrophils and are important profibrotic factors (Bellaye et al., 2018; Matin et al., 2018). Recent studies have found that IL-17 is an important profibrotic factor (Zhang et al., 2019).

Exercise is a tool to treat many autoimmune diseases, including COPD, asthma and pulmonary fibrosis, because AE has immunomodulatory effects and modulates the redox balance (Holland and Hill, 2008; Nishiyama et al., 2008; Vainshelboim et al., 2014; Otoupalova et al., 2020). Here, we attempt to demonstrate whether an 8-week AE programme can improve HFD-induced pulmonary fibrosis in obese mice and to explore the related mechanisms: insulin resistance, inflammation, and oxidative stress.

The lung-protective effects of sirt-1 are well documented (Nakamaru et al., 2009; Rahman et al., 2012). Sirt-1 can regulate the transcriptional control of multiple genes related to anti-inflammatory action, antioxidation, and energetic metabolism (Rajendrasozhan et al., 2008; Yao et al., 2012). Sirt-1, which can be activated by exercise, improves pulmonary inflammation and modulates the pro-oxidative/antioxidative balance in the lung (Cantó et al., 2009). Sirt-1 was significantly associated with lung protection. AE may activate Sirt-1 expression in obese mice and therefore counter obesity-associated pulmonary fibrosis.

AE provides insight into pulmonary fibrosis, while the underlying molecular mechanisms through which AE protects against HFD-induced pulmonary fibrosis require more research.

## Materials and Methods

### Animal and Experimental Groups

All protocols used in this study were approved the Animal Experimental Welfare of the Institute of Animal Science, Chinese Academy of Agricultural Sciences (Beijing, China). All experiments were performed in accordance with the Animal Experimental Welfare of the Institute of Animal Science, Chinese Academy of Agricultural Sciences and the Guide for the Care and Use of Laboratory Animals published by the US National Institutes of Health. The mice were anaesthetized *via* intraperitoneal injection of pentobarbital (50 mg/kg).

Forty-eight male C57BL/6 mice were randomly divided into four groups: (1) chow (Ch) group, where the mice were fed a chow diet for 16 weeks; (2) chow diet plus exercise (CE) group, where the mice were fed a chow diet for 16 weeks and forced to train in the last 8 weeks; (3) the obesity (Ob) group, where the mice were fed a HFD for 16 weeks; and (4) the obesity plus exercise (OE) group, where the mice were fed a HFD for 16 weeks and forced to train in the last 8 weeks. Each group included 12 mice.

### Exercise Protocol

OE mice were forced to train using a treadmill. Treadmill aerobic training lasted for 8 weeks and was performed once a day for 60 min per session (0^°^ incline, 18 m/min).

### Sample Collection

Blood samples were collected from the mice and placed into blood collection vessels. After centrifugation, the upper serum layer was harvested and frozen at −20°C immediately. The remaining lung tissues were frozen with liquid nitrogen.

Two millilitres of 0.9% normal saline was utilised for whole-lung lavage. The whole lung was flushed five times. The BALF was centrifuged at 3,500 r/min for 20 min. The BALF was immediately stored at −80°C.

### Histopathology

Tissue samples were fixed with 4% paraformaldehyde. Five-micrometre thick sections were obtained using the paraffin section method. Haematoxylin and eosin staining were utilised to detect the degree of inflammation in the lung. Masson and Sirius Red staining were utilised to detect collagen fibre deposition in the lung. Images were captured with an inverted microscope.

### Determination of Pulmonary Inflammation

BALF levels of CXCL-1, IL-1β, IL-10, IL-17, TNF-α, and TGF-β were detected with commercial kits (Multi sciences (Lianke) Biotech, CO., LTD, Hangzhou, China).

### Determination of Oxidative Stress Index in Lung Homogenates

Following the manufacturer’s specifications, superoxide dismutase (SOD), malondialdehyde (MDA), myeloperoxidase (MPO), and glutathione (GSH) levels in the mice were detected using spectrophotometry as previously described (Bio-Tek Instruments Inc., software KC4 v3.0) (Feng et al., 2016; Mai et al., 2021). We measured the conjugation of 1-chloro-2,4-dinitrobenzene (CDNB) to evaluate GST activity. The Bradford Method was used to evaluate MDA activity.

### Determination of mRNA Expression Levels

Total RNA was extracted from DNase I-treated cells using TRIzol as previously described (Broderick et al., 2017). Total RNA (2.0 μg) was used as a template for cDNA synthesis. Real-time fluorescence quantitative PCR was used to measure gene expression levels. GAPDH served as the housekeeping gene. All primers were designed and synthesised by Shanghai Sangon Biotech Company. Table 1 shows the murine PCR primer sequence information.

**TABLE 1:** PCR primer sequence information.

Gene name	Primer sequence
CXCL-1	ACCTTCATCGGAAACTCCAAAG
	CTGTTAGGCTGGGAAAAGTTAGG
IL-1β	CTGCCGTCCGATTGAGACC
	CCCCTCCTTGTACCACTGTC
IL-10	CAAACCTCAATGTGTCTCTTTGC
	AGAGTAAAGCCTATCTCGCTGT
IL-17	CGAAGCGTGTGAAGGCAAC
	TTGTACGGGCCTGACATTTCC
MMP-9	ACATCGACCCGTCCACAGTAT
	CAGAGGGGTAGGCTTGTCTC
Sirt-1	CTCCCAACAGA CCTGTCTATAC
	CCATTGCACAACTCTTTTCTCA
TGF-β	AAACAGATGAAGTGCTCCTTCCAGG
	TGGAGAACACCACTTGTTGCTCCA
TNF-α	ATGTCTCAGCCTCTTCTCATTC
	GCTTGTCACTCGAATTTTGAGA
GAPDH	AGGTCGGTGTGAACGGATTTG
	TGTAGACCATGTAGTTGAGGTCA

*CXCL, C-X-C motif chemokine ligand; IL, interleukin; MMP, matrix metalloproteinase; PCR, polymerase chain reaction; Sirt, sirtuin; TGF, transforming growth factor; TNF, tumour necrosis factor.*

### Detection of Insulin Resistance

According to the reagent manufacturer’s specifications, plasma glucose levels were detected with a glucometer (Lifescan Benelux, Beerse, Belgium). The plasma insulin and adiponectin levels were detected using commercial kits (Shino Test Corporation, Tokyo, Japan).

### Data Processing

All data were expressed as the mean ± SEM. Two-way ANOVA and Tukey’s *post hoc* test was utilised to analyse the data in this study. The significance threshold was set at *P* < 0.05. GraphPad Prism 9 software was utilised to analyse the data and draw figures.

## Results

### Effects of Aerobic Exercise on High-Fat Diet-Induced Adiposity

As Table 2 shows, untrained obese mice had higher body weights (*P* < 0.001), net body weight gains (*P* < 0.001), abdominal fat weights (*P* < 0.001), and subcutaneous fat weights (*P* < 0.001) than did Ch mice. OE mice had lower body weights (*P* < 0.001), net body weight gains (*P* < 0.001), abdominal fat weights (*P* < 0.001), and subcutaneous fat weights (*P* < 0.001) than did Ob mice.

**TABLE 2:** Effects of HFD and exercise on body mass and body composition.

Group	Con	Ex	Ob	OE
Initial body weight (g)	20.62 ± 2.28	20.33 ± 2.81	20.24 ± 2.03	20.55 ± 2.94
Final body weight (g)	30.43 ± 5.68	26.11 ± 3.68	44.4 ± 6.28*	35.49 ± 4.97^#^
Body weight gain (g)	9.81 ± 1.08	5.78 ± 0.62	24.16 ± 3.28*	14.94 ± 1.37^#^
Subcutaneous fat weight (g)	0.28 ± 0.04	0.2 ± 0.03	1.88 ± 0.31*	0.95 ± 0.15^#^
Subcutaneous fat/body weight ratio (%)	0.93 ± 0.13	0.76 ± 0.08	4.23 ± 0.68*	2.68 ± 0.43^#^
Abdominal fat weight (g)	1.35 ± 0.24	0.95 ± 0.16	4.62 ± 0.86*	2.58 ± 0.51^#^
Abdominal/body weight ratio (%)	4.45 ± 0.52	3.64 ± 0.33	10.42 ± 1.34*	7.26 ± 0.97^#^

^#^P < 0.05 compared with the Ch group. *P < 0.05 compared with the Ob group.

### Detection of Pulmonary Inflammation

**FIGURE 1:**
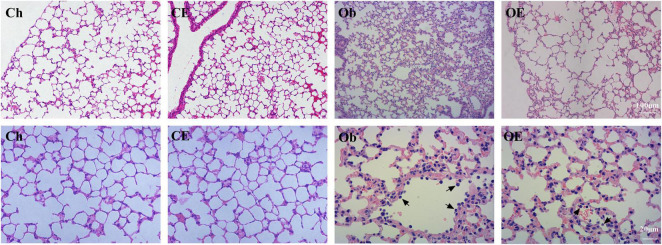
Morphological analysis of lung tissue. Haematoxylin-eosin staining revealed evidence of the degree of inflammatory cell infiltration. Black arrows indicate neutrophils.

In the Ch group and the CE group, no inflammatory cell infiltration was observed in the lung. HFD markedly increased the number of neutrophils in the lung, while AE markedly decreased the number of neutrophils in obese mice (Figure 1).

As shown in Table 3, after HFD feeding for 16 weeks, the number of neutrophils (*P* < 0.001) in BALF was markedly upregulated. Regular AE markedly downregulated the number of neutrophils in obese mice (*P* = 0.0301).

**TABLE 3:** Total and differential cell counts in BALF (cells/ml).

Group	Con	Ex	Ob	OE
Total cells	1.29 ± 0.86	1.18 ± 0.75	3.54 ± 0.41*	2.14 ± 0.34^#^
Neutrophils	0.04 ± 0.01	0.04 ± 0.01	0.55 ± 0.093*	0.34 ± 0.088^#^
Lymphocytes	0.03 ± 0.05	0.02 ± 0.04	0.04 ± 0.14	0.03 ± 0.06
Macrophages	1.09 ± 0.38	0.98 ± 0.22	2.57 ± 0.77*	1.56 ± 0.57^#^
Eosinophils	0.01 ± 0.002	0.01 ± 0.003	0.02 ± 0.01	0.02 ± 0.006

*The number of total cells, neutrophils, eosinophils, and macrophages in BALF were detected.*

*P < 0.05 for difference from Ch; ^#^P < 0.05 for difference from Ob.

As shown in Table 4, untrained obese mice had significantly higher levels of the neutrophil chemokines TNF-α (*P* < 0.001) and CXCL-1 (*P* < 0.001), profibrogenic factors TGF-β (*P* < 0.001) and IL-17 (*P* < 0.001), and proinflammatory factor IL-1β (*P* < 0.001) but significantly lower levels of the anti-inflammatory factor IL-10 (*P* < 0.001) in BALF than did Ch mice. AE markedly decreased BALF levels of IL-1β (*P* = 0.033), IL-17 (*P* = 0.009), TGF-β (*P* = 0.013), and TNF-α (*P* = 0.025) but markedly increased BALF levels of IL-10 (*P* = 0.004) compared with Ob mice.

**TABLE 4:** The levels of inflammatory factors in BALF (pg/ml).

Group	Con	Ex	Ob	OE
CXCL-1	8.56 ± 1.76	8.01 ± 1.13	43.68 ± 10.62*	25.34 ± 5.94^#^
IL-17	8.23 ± 1.97	7.88 ± 0.84	53.56 ± 13.89*	31.56 ± 7.82^#^
TGF-β	20.34 ± 4.86	18.11 ± 2.43	64.42 ± 15.45*	41.32 ± 10.58^#^
IL-10	15.34 ± 6.72	35.49 ± 4.29*	5.12 ± 2.45*	26.45 ± 4.83^#^
IL-1β	17.26 ± 2.14	15.65 ± 0.18	46.89 ± 10.67*	24.36 ± 5.84^#^
TNF-α	16.54 ± 4.81	14.22 ± 1.59	59.34 ± 16.47*	46.89 ± 13.56^#^

*The BALF levels of CXCL-1, IL-17, TGF-β, IL-10, IL-1β, and TNF-α were detected.*

^#^P < 0.05 compared with the Ch group. *P < 0.05 compared with the Ob group. CXCL, C-X-C motif chemokine ligand; IL, interleukin; TGF, transforming growth factor; TNF, tumour necrosis factor.

### Detection of Pulmonary Fibrosis

**FIGURE 2:**
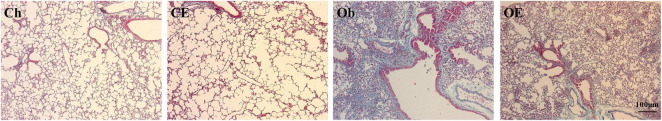
Collagen deposition was detected by Masson staining. Masson staining revealed evidence of the degree of pulmonary fibrosis in the lung.

Masson staining showed the degree of pulmonary fibrosis. Compared with those in the Ch group and the CE group, the magnitude of pulmonary fibrosis increased after 16 weeks of HFD administration, but AE decreased the magnitude of pulmonary fibrosis in obese mice (Figure 2).

**FIGURE 3:**
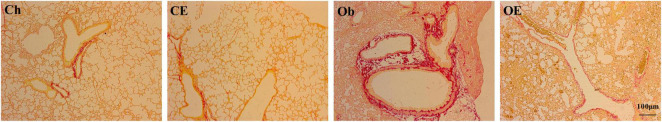
Collagen deposition was detected by Sirius Red staining. Sirius Red staining revealed evidence of collagen fibre deposition in the lung.

Sirius Red staining showed collagen fibre deposition in lung tissue. In the Ch group and the CE group, no obvious collagen fibre deposition was noted in the airway wall, while collagen fibre deposition in the airway wall increased after 16 weeks of HFD administration. AE decreased collagen fibre deposition in the airway wall in obese mice (Figure 3).

As shown in Table 4, HFD administration markedly upregulated the profibrogenic factors TGF-β (*P* = 0.024) and IL-17 (*P* = 0.0031) in BALF. Regular AE downregulated BALF levels of TGF-β (*P* = 0.013) and IL-17 (*P* = 0.003) in obese mice.

As Supplementary Material 1 showed, HFD administration markedly upregulated hydroxyproline levels (*P* < 0.001), Ashcroft fibrosis (*P* < 0.001), lung fibrotic score (*P* < 0.001), and airway collagen (*P* < 0.001) in the lung, whereas AE markedly downregulated hydroxyproline levels (*P* = 0.019), Ashcroft fibrosis (*P* = 0.024), lung fibrotic score (*P* = 0.031), and airway collagen (*P* = 0.005) in obese mice.

### Effects of Aerobic Exercise on mRNA Expression Levels in the Lung

**FIGURE 4:**
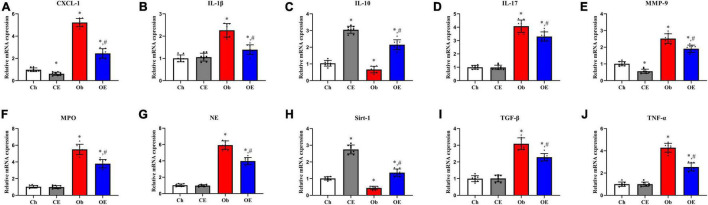
Detection of mRNA expression levels in lung tissue. Detection of mRNA expression levels of CXCL-1 **(A)**, IL-1β **(B)**, IL-10 **(C)**, IL-17 **(D)**, MMP-9 **(E)**, MPO **(F)**, NE **(G)**, sirt-1 **(H)**, TGF-β **(I)**, and TNF-α **(J)** in lung tissue. ^#^*P* < 0.05 compared with the Ch group. *P < 0.05 compared with the Ob group. CXCL, C-X-C motif chemokine ligand; IL, interleukin; MMP, matrix metalloproteinase; NE, neutrophil elastase; Sirt, sirtuin; TGF, transforming growth factor; TNF, tumour necrosis factor.

The mRNA expression levels of CXCL-1 (Figure 4A), IL-1β (Figure 4B), IL-10 (Figure 4C), IL-17 (Figure 4D), MMP-9 (Figure 4E), MPO (Figure 4F), NE (Figure 4G), sirt-1 (Figure 4H), TGF-β (Figure 4I), and TNF-α (Figure 4J) in the lung were detected. HFD feeding upregulated CXCL-1 (*P* < 0.001), IL-1β (*P* < 0.001), IL-17 (*P* = 0.0034), MMP-9 (*P* = 0.0089), MPO (*P* < 0.001), NE (*P* < 0.001), TGF-β (*P* < 0.001), and TNF-α (*P* = 0.0072) mRNA expression levels but decreased IL-10 (*P* = 0.013) and sirt-1 (*P* = 0.011) mRNA expression levels compared with the Ch group. AE for 8 weeks markedly downregulated CXCL-1 (*P* < 0.001), IL-1β (*P* < 0.001), IL-17 (*P* = 0.024), MMP-9 (*P* = 0.0029), MPO (*P* = 0.013), NE (*P* = 0.011), TGF-β (*P* = 0.0125), and TNF-α (*P* < 0.001) mRNA expression levels in obese mice.

### Detection of Oxidative Stress Index in the Lung

**FIGURE 5:**
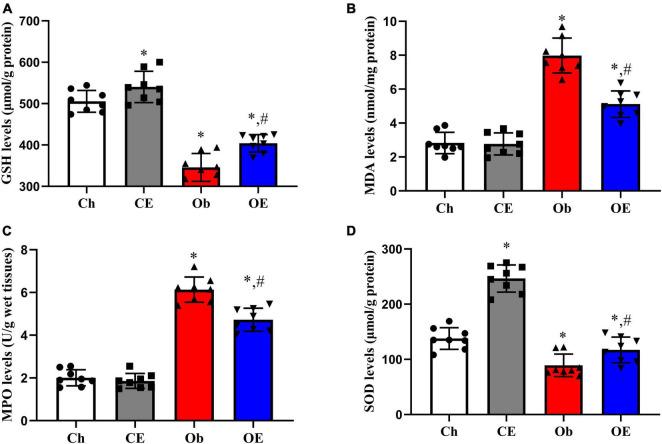
Detection of oxidative stress-related genes in lung tissue. The levels of GSH **(A)**, MDA **(B)**, MPO **(C)**, and SOD **(D)** in lung tissue were detected. ^#^*P* < 0.05 compared with the Ch group. *P < 0.05 compared with the Ob group. MDA, malondialdehyde; MPO, myeloperoxidase; GSH, glutathione; SOD, superoxide dismutase.

The levels of GSH (Figure 5A), MDA (Figure 5B), MPO (Figure 5C), and SOD (Figure 5D) in the lung were detected. HFD feeding resulted in decreased expression levels of GSH (*P* = 0.0063) and SOD (*P* = 0.0047) and increased expression levels of MDA (*P* = 0.0092) and MPO (*P* = 0.0025) in lung tissue compared with the Ch group. Significant downregulation of MPO (*P* = 0.0396) and MDA (*P* = 0.0412) expression and significant upregulation of GSH (*P* = 0.0037) and SOD (*P* = 0.0268) expression were detected after 8 weeks of AE compared with the Ob group.

### Effects of Aerobic Exercise on High-Fat Diet-Induced Insulin Resistance

**FIGURE 6:**
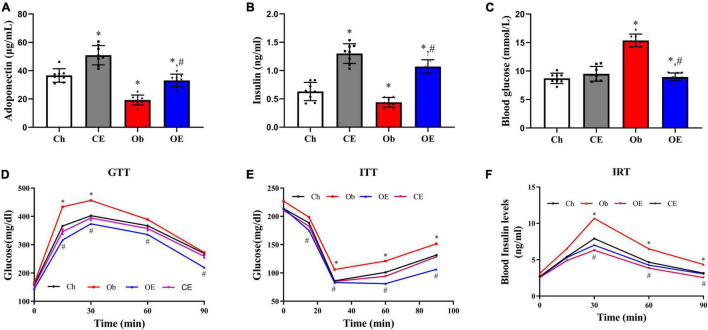
Detection of insulin resistance. **(A)** Plasma adiponectin levels, **(B)** plasma insulin levels, **(C)** blood glucose levels, **(D)** GTT, **(E)** ITT, **(F)** IRT. ^#^*P* < 0.05 compared with the Ch group. *P < 0.05 compared with the Ob group. GTT, glucose tolerance test; ITT, insulin tolerance test; IRT, insulin release test.

HFD feeding markedly downregulated plasma adiponectin levels (Figure 6A; *P* < 0.001) and plasma insulin levels (Figure 6B; *P* < 0.001) but markedly upregulated blood glucose levels (Figure 6C; *P* < 0.001) compared with Ch treatment. Significant downregulation of blood glucose levels (*P* = 0.0164) and significant upregulation of plasma adiponectin levels (*P* = 0.0386) and plasma insulin levels (*P* = 0.0441) were detected after AE for 8 weeks. The results of the GTT (Figure 6D), ITT (Figure 6E), and IRT (Figure 6F) demonstrated that an 8-week AE programme in obese mice markedly improved insulin resistance.

## Discussion

Previous studies demonstrate that obesity was closely associated with pulmonary fibrosis (Murtha et al., 2017; Rathinasabapathy et al., 2018; Han et al., 2021). However, the protective effects of AE on pulmonary fibrosis were unclear. Our data demonstrated that AE in obese mice had an antifibrotic effect and significantly decreased the magnitude of pulmonary fibrosis.

Morphological analysis demonstrated that AE in obese mice reduced hydroxyproline levels and collagen fibre deposition in lung tissue. Our data demonstrated that HFD administration dramatically increased the levels of the profibrogenic factors NE, MMP-9, TGF-β, and IL-17 in BALF. Similarly, HFD administration dramatically increased NE, MMP-9, TGF-β, and IL-17 mRNA expression levels in lung tissue, which was reversed by the end of the 8-week AE programme. Thus, AE protected against pulmonary fibrosis by repressing the expression of NE, MMP-9, TGF-β, and IL-17. In addition, sirt-1 was a negative regulator of MMP-9 (Nakamaru et al., 2009). Therefore, AE may suppress MMP-9 levels in part by activating sirt-1 levels in the lung.

Neutrophils play an important role in the pathogenesis of pulmonary fibrosis (Stockley, 2002; Jackson et al., 2014). We found that regular AE decreased the number of neutrophils in BALF in obese mice. Thus, AE reduced pulmonary fibrosis in part by reducing the neutrophil content in the lung. Previous studies have demonstrated that CXCL-1 and TNF-α are chemotactic for neutrophils (Thannickal et al., 2004; Matin et al., 2018). Our data demonstrated that regular AE in obese mice markedly suppressed BALF levels of CXCL-1 and TNF-α. Our data showed that regular AE in obese mice markedly suppressed the mRNA expression levels of TNF-α and CXCL-1 in the lung. Hence, AE inhibited neutrophil infiltration in part by suppressing CXCL-1 and TNF-α expression.

HFD administration led to pro-oxidative/antioxidative imbalance, and the antioxidant effects of exercise were identified in this study. Our data identified that regular AE in obese mice significantly downregulated MDA and MPO levels but significantly upregulated SOD and GSH levels in the lung. AE in obese mice modulated the oxidative/antioxidative balance in lung tissue. Previous studies have demonstrated that sirt-1 has therapeutic effects in lung diseases because sirt-1 modulates the pro-oxidative/antioxidative balance (Rajendrasozhan et al., 2008; Nakamaru et al., 2009; Rahman et al., 2012). Our data showed that AE modulated the oxidation/antioxidation balance in lung tissues partly because AE increased sirt-1 expression. Sirt-1 exerts anti-inflammatory and antioxidant effects and protects against lung diseases, including COPD, asthma, and pulmonary fibrosis (Cantó et al., 2009; Yao et al., 2012). Thus, obesity increases the probability of lung diseases, including COPD and pulmonary fibrosis, in part by suppressing sirt-1 production. Therefore, targetted improvement of sirt-1 production in obese patients with pulmonary fibrosis may be a novel therapeutic strategy.

Previous studies identified that obesity-associated insulin resistance enhanced TGF-β expression, which played an important role in the pathogenesis of pulmonary fibrosis (Spencer et al., 2010; Mayer et al., 2012; Asghar and Sheikh, 2017; Park et al., 2019). We found that AE improved HFD-induced insulin resistance and decreased TGF-β mRNA expression in the lung and BALF levels of TGF-β. Hence, AE alleviated pulmonary fibrosis by improving insulin resistance and suppressing TGF-β expression. Exercise is Medicine (Li and Laher, 2015, 2020; Li et al., 2019).

### Perspective

The results of this study provide insight into pulmonary fibrosis and indicate that AE is a novel tool to treat pulmonary fibrosis. HFD administration leads to pulmonary fibrosis by causing insulin resistance, a chronic inflammatory response, pro-oxidative/antioxidative imbalance, and increased levels of profibrogenic factors, including TGF-β, IL-17, NE, and MMP-9. AE improves HFD-induced pulmonary fibrosis by counteracting obesity-associated insulin resistance, chronic inflammatory responses, and pro-oxidative/antioxidative imbalance and shows an antifibrogenic effect by suppressing TGF-β, IL-17, NE, and MMP-9 production in obese mice.

## Data Availability Statement

The original contributions presented in the study are included in the article/Supplementary Material, further inquiries can be directed to the corresponding authors.

## Ethics Statement

The animal study was reviewed and approved by Animal Experimental Welfare and Ethical Inspection Form of Institute of Animal Science, Chinese Academy of Agricultural Sciences.

## Author Contributions

XW: conceptualisation, methodology, software, validation, writing—original draft preparation, and project administration. XY and DT: formal analysis, investigation, resources, supervision, and funding acquisition. XW, XY, and DT: writing—review and editing. All authors have read and agreed to the published version of the manuscript.

## Conflict of Interest

The authors declare that the research was conducted in the absence of any commercial or financial relationships that could be construed as a potential conflict of interest.

## Publisher’s Note

All claims expressed in this article are solely those of the authors and do not necessarily represent those of their affiliated organizations, or those of the publisher, the editors and the reviewers. Any product that may be evaluated in this article, or claim that may be made by its manufacturer, is not guaranteed or endorsed by the publisher.
