# Supplementary material for: Aerobic Exercise Improves Pulmonary Fibrosis by Improving Insulin Resistance and Inflammation in Obese Mice
Source: Front Physiol. 2022 Jan 18;12:785117. doi: 10.3389/fphys.2021.785117 (PMC8804531; doi:10.3389/fphys.2021.785117)
Supplement: Supplementary file 1 [file Data_Sheet_1.docx]

**Supplementary Material 1**.

**BALF cell count:** The numbers of total cells, neutrophils, lymphocytes, eosinophils, and macrophages in BALF were measured. Total and differential cell counts in bronchoalveolar lavage fluid (BALF) were measured [1,2]. Blood was collected through the abdominal vein, and BALF was collected by flushing the lungs three times with 0.5 ml of 37 °C sterile, pyrogen-free, physiological saline (0.9% NaCl) via the tracheal cannula. After BALF collection, the samples were centrifuged at 420 × g at 4 °C for 15 minutes, and the supernatant was stored at -70 °C for subsequent analysis of protein and cytokine levels. The cell pellets were resuspended in 1 ml of PBS, and total cell counts were performed using a Neubauer’s chamber. For differential cell counts, cytospin slides were prepared and stained with Diff-Quick; 300 cells were counted per slide.

**Neutrophil content:** By using a 100-point grid of a known area (62,500 μm^2^ at 400X magnification) attached to the ocular lens of the microscope, we counted the number of points contacting the tissue samples and the numbers of neutrophils and positive cells in each field. The cell density was determined as the number of positive cells in each field divided by the tissue area, and this value was expressed as cells/mm^2^ [3]. Morphometric measurements were performed in 15 fields for each animal at 400X magnification by an investigator who was blinded to the experimental group.

specific group under study.

**Lung injury score:** Lung tissues were subjected to paraffin procedures, sectioned at approximately 5-μm thickness, and then stained with HE. Lung injury was evaluated and scored by two pathologists who were blinded to the experimental design by using a recent criterion [4], where lung damage was evaluated on a two-point scale with scores ranging from 0 to 1.

**Detection of pulmonary fibrosis:** To assess alveolitis and fibrosis, Masson-stained sections were evaluated via semiquantitative histology by a pathologist who was blinded to the treatment groups using a light microscope and a scoring system for measurements according to a previously described method [5,6]. A histological semiquantitative examination of the lung was performed on sections after standard Sirius Red staining. Periportal fibrosis was scored 0-4, and perisinusoidal fibrosis was scored 0-2, thus yielding a maximum possible score of 6 [7].

**Hydroxyproline Assay:** The lung tissues were homogenized in 10 volumes of distilled water. Homogenates were hydrolysed in 12 M HCl at 120 °C for 3 h as previously described [8]. Hydroxyproline was measured colorimetrically (560 nm) after hydrolysis using an assay kit (Nanjing Jiancheng Bioengineering Institute, Nanjing, China).

**Ashcroft fibrosis:** After death, each lung tissue was fixed in buffered 4% paraformaldehyde for 24 hr and embedded in paraffin. Ten consecutive longitudinal sections of the lungs were stained with haematoxylin and eosin (HE) and examined for pulmonary fibrosis. Each successive field was individually assessed for the severity of interstitial fibrosis using the semiquantitative grading system described by Ashcroft et al. [9]. The entire lung section was reviewed at a magnification of 100. For each of the 30-35 microscopic fields needed to review the section, a score ranging from 0 (normal lung) to 8 (total fibrosis) was assigned. The mean score of all fields was taken as the fibrosis score of that lung section. The criteria for grading pulmonary fibrosis were as follows. Grade 0 ¼ was assigned to a normal lung; Grade 1 ¼ was assigned to a minimal fibrous thickening of alveolar or bronchial walls; Grade 2-3 ¼ was assigned to a moderate thickening of walls without obvious damage to lung architecture; Grade 4-5 ¼ was assigned to an increased fibrosis with definite damage to lung architecture and formation of fibrous bands or small fibrous mass; Grade 6-7 ¼ was assigned to severe distortion of structure and large fibrous areas, and honeycomb lung was placed in this category; and Grade 8 ¼ was assigned to a total fibrotic obliteration of the field.

| **Group** | **Ch** | **CE** | **Ob** | **OE** |
| --- | --- | --- | --- | --- |
| Hydroxyproline (μg/μng) | 15.11 ± 1.49 | 14.89 ± 1.24 | 34.22 ± 3.16^*^ | 23.65 ± 2.41^#^ |
| Ashcroft fibrosis | 0.32 ± 0.03 | 0.29 ± 0.02 | 4.56 ± 0.46^*^ | 3.38 ± 0.23^#^ |
| Lung fibrotic score | 1.01 ± 0.09 | 0.98 ± 0.08 | 3.22 ± 0.37^*^ | 1.76 ± 0.15^#^ |
| Airway collagen (μm^2^ collagen/μm^2^ tissues) | 20.66 ± 2.14 | 18.31 ±1.92 | 87.24 ± 0.95^*^ | 40.55 ±0.58^#^ |

**Notes:** ^#^ *P*<0.05 compared with the Ch group. ^*^ *P*<0.05 compared with the Ob group.

**References**

[1] Vieira RP, Claudino RC, Duarte AC, et al. Aerobic exercise decreases chronic allergic lung inflammation and airway remodeling in mice. Am J Respir Crit Care Med. 2007; 176(9):871-7. DOI: 10.1164/rccm.200610-1567OC.

[2] Kasahara DI, Poynter ME, Othman Z, et al. Acrolein inhalation suppresses lipopolysaccharide-induced inflammatory cytokine production but does not affect acute airways neutrophilia. J Immunol. 2008; 181(1):736-45. doi: 10.4049/jimmunol. 181.1.736.

[3] Lanças T, Kasahara DI, Prado CM, et al. Comparison of early and late responses to antigen of sensitized guinea pig parenchymal lung strips. J Appl Physiol. 2006;100:1610-1616. doi: 10.1152/ japplphysiol.00828.2005.

[4] Gustavo Matute-Bello, Gregory Downey, Bethany B Moore, et al. An official American Thoracic Society workshop report: features and measurements of experimental acute lung injury in animals. Am J Respir Cell Mol Biol. 2011;44(5):725-38. doi: 10.1165/rcmb.2009-0210ST.

[5] Szapiel SV, Elson NA, Fulmer JD, et al. Bleomycin-induced interstitial pulmonary disease in the nude, athymic mouse. Am Rev Respir Dis. 1979;120:893‑899. doi: 10.1164/arrd.1979.120.4.893.

[6] Fulmer JD, Bienkowski RS, Cowan MJ, et al. Collagen concentration and rates of synthesis in idiopathic pulmonary fibrosis. Am Rev Respir Dis. 1980;122: 289-301. doi: 10.1164/arrd.1981.124.3.341a.

[7] Sebastian Huss, Jörg Schmitz, Diane Goltz, et al. Development and evaluation of an open source Delphi-based software for morphometric quantification of liver fibrosis. Fibrogenesis Tissue Repair. 2010; 17;3(1):10. doi: 10.1186/1755-1536-3-10.

[8] Kennedy J, Chandler D, Jackson R, Fulmer J. Reduction in bleomycin- induced lung hydroxyproline content by an iron chelating agent. Chest. 1986;89(3 Suppl):123S-125S. doi: 10.1378/chest.89.3_Supplement.123S.

[9] Yildirim Z, Kotuk M, Erdogan H, et al. Preventive effect of melatonin on bleomycin-induced lung fibrosis in rats. J Pineal Res. 2006;40(1):27-33. doi: 10. 1111/j.1600-079X.2005.00272.x.
